# Natriuretic peptides modulate monocyte-derived Langerhans cell differentiation and promote a migratory phenotype

**DOI:** 10.3389/fimmu.2025.1593141

**Published:** 2025-06-09

**Authors:** Dorottya Horváth, Zsófia Pénzes, Petra Molnár, István Rebenku, György Vereb, Magdolna Szántó, Szabolcs Muzsai, Andrea Szegedi, Zsolt Dajnoki, Kitti Pázmándi, Tünde Fekete, Attila Bácsi, Attila Gábor Szöllősi

**Affiliations:** ^1^ Department of Immunology, Faculty of Medicine, University of Debrecen, Debrecen, Hungary; ^2^ Doctoral School of Molecular Medicine, University of Debrecen, Debrecen, Hungary; ^3^ Department of Biophysics and Cell Biology, Faculty of Medicine, University of Debrecen, Debrecen, Hungary; ^4^ HUN-REN-UD Cell Biology and Signaling Research Group, Faculty of Medicine, University of Debrecen, Debrecen, Hungary; ^5^ Faculty of Pharmacy, University of Debreen, Debrecen, Hungary; ^6^ Department of Medical Chemistry, Faculty of Medicine, University of Debrecen, Debrecen, Hungary; ^7^ Gyula Petrányi Doctoral School of Clinical Immunology and Allergology, University of Debrecen, Debrecen, Hungary; ^8^ Department of Dermatology, Centre of Excellence, Faculty of Medicine, University of Debrecen, Debrecen, Hungary; ^9^ HUN-REN-UD, Allergology Research Group, Faculty of Medicine, Debrecen, Hungary

**Keywords:** monocyte-derived Langerhans cell, neuropeptides, atrial natriuretic peptide, B-type natriuretic peptide, calcitonin gene related peptide, neurotensin, skin immunology, atopic dermatitis

## Abstract

**Introduction:**

The interaction between the nervous and immune systems is crucial for maintaining homeostasis and can influence disease progression in inflammatory skin diseases, such as atopic dermatitis (AD). Sensory neurons in the skin can secrete neuropeptides that modulate immune cell activity, including Langerhans cells (LCs), one of the primary antigen-presenting cells in the epidermis. In our study we investigated the effects of neuropeptides on the differentiation of monocyte-derived LCs (moLCs), specifically the neuropeptides with the most profound effect, i.e. atrial- and B-type natriuretic peptides (ANP and BNP, respectively).

**Methods:**

RNA sequencing and RT-qPCR were used to analyze neuropeptide receptor expression in moLCs and immature dendritic cells (iDCs), and the most translationally relevant, natriuretic peptide receptor A (NPR1) was validated on the protein level using western blotting. Cell surface markers of moLCs were assessed using flow cytometry, and NPR1 functionality was confirmed through intracellular cGMP assays. Confocal microscopy was used to confirm the expression of NPR1 *in situ* in healthy and AD skin. RNA-Seq analysis was also employed to characterize the phenotypic changes in moLCs differentiated in the presence of BNP.

**Results:**

NPR1 expression was significantly higher in moLCs compared to iDCs, and treatment with ANP and BNP enhanced moLC differentiation, increasing CD207, CD1a, and HLA-DQ expression, while other tested neuropeptides (calcitonin gene-related peptide [CGRP], neurotensin) had no significant effect. NPR1 was functionally active, as evidenced by increased intracellular cGMP levels upon ligand binding. Confocal microscopy revealed NPR1 expression on LC cell bodies in both healthy and AD skin, with reduced intensity in AD. RNA-Seq analysis of BNP-treated moLCs indicated a shift toward a migratory LC phenotype, marked by upregulation of genes associated with cell motility (e.g., CCR7, LAMP3).

**Discussion:**

These findings demonstrate that NPR1 activation promotes a migratory LC phenotype, highlighting the role of neuropeptides in shaping cutaneous immune responses. The reduced number of LCs in AD skin suggests a potential link between neuropeptide signaling and disease pathology.

## 1 Introduction

The interaction between the immune and nervous systems is important in maintaining homeostasis during normal immunological processes, and also in the development of disease. Sensory afferent neurons create a functionally and phenotypically diverse network in the skin. They can be classified based on their morphology, the stimuli they are most sensitive to, and also based on the mediators they produce during signal transduction (1, 2). On top of their afferent function, i.e. the transduction of sensory information towards the central nervous system, neurons also communicate locally with surrounding cells by secreting different signal molecules such as fatty acids, amino acids, gases, and proteins. One of the most prominent locally produced mediators are small, nerve-derived peptide signaling molecules, commonly called neuropeptides (3, 4). Neuropeptides are some of the most diverse families of these signaling molecules. Neuropeptides can act in an autocrine or paracrine ways, but they are also viable in long distances (3–5). They can cause neuroimmune inflammation by activating adaptive and innate immune cells, as well as dampening immune activation depending on the molecular context present (6–9).

Several neuropeptides are present in normal human skin and participate in cutaneous functions and diseases including calcitonin gene-related peptide (CGRP), neurotensin, vasoactive intestinal peptide, substance P, neurokinin A, neuropeptide Y, atrial natriuretic peptide (ANP) (5, 10–14). Another neuropeptide from the natriuretic peptide family, B-type natriuretic peptide (BNP), is a key mediator of itch both in the periphery and the central nervous system. In atopic dermatitis (AD), BNP is released from skin peripheral nerve endings after IL-31 secretion by Th2 cells, and contributes to itch formation, and activates keratinocytes to increase IL17A, CXCL10, and MMP9 production and acts on dendritic cells (DCs) to produce CCL20 (15).

In the skin, neurons modulate immune cell functions with neuropeptide secretion in response to both pathogens and tissue damage. For example, *Streptococcus pyogenes* can block neutrophil granulocytes at the site of infection through neurons. Sensory neurons triggered by streptolysin S produce CGRP, which inhibits both neutrophil recruitment and activation (16). *Candida albicans* activates sensory neurons to secrete CGRP, which contributes to dermal DCs’ (DDCs) IL-23 production and this effect regulates γδ T cells’ IL-17 secretion (17). Furthermore, substance P released from house dust mite-activated nociceptors can drive allergic inflammation by inducing mast cell degranulation (18).

This immunomodulatory effect is not only present during infections but also in some inflammatory skin diseases, such as AD and psoriasis, which are known to be exacerbated by psychological stress (19). Psychological stress has been shown to result in increased numbers of peptidergic nerve fibers (20), and increased substance P release from these fibers (21–23), which is also detectable in the serum of mice exposed to chronic stress (24).

Supporting the direct link between the nervous system and these inflammatory skin diseases some clinical case reports show that skin lesions cannot fully develop in nerve-injured areas in the skin, and only return once innervation is reestablished (25–31).

A likely target of epidermally released neuropeptides are Langerhans cells (LCs), which are believed to be the sole professional antigen-presenting immune cells under steady-state in the epidermal layer of human skin until recent studies that show a CD11c+ epidermal dendritic cell population (32, 33). Coincidentally, LCs are often anatomically associated with CGRP+ nerve endings (34–36). Epidermal LCs and DCs express Langerin (CD207), a C-type lectin receptor, as well as CD1a. The absence of CD206 expression, decreased expression of CD1c, CD11b, and CD11c, and the presence of Birbeck granules are characteristics that distinguish epidermal LCs from DCs (32, 37). Although ontogenetically they are similar to tissue-resident macrophages, as they originate from macrophage precursors in the embryonic yolk sac, functionally LCs are more similar to DCs, since they can migrate to draining lymph nodes to present antigens to naïve T cells. To support homeostasis in the skin LCs have immunogenic and tolerogenic functions depending on the immune response (38). Although LCs are long-lived and have self-renewing capability, they can also differentiate from bone marrow-derived monocytes following major inflammatory skin injuries (39).

Although numerous protocols have been published in the literature to study monocyte-derived LCs (moLCs), there are no optimal cytokine cocktails and differentiation conditions that are widely accepted (40–42). Prior research has demonstrated that LC-like cells can be differentiated *in vitro* using granulocyte/macrophage colony-stimulating factor (GM-CSF), IL-4, and transforming growth factor β (TGF-β) from CD14^+^ monocytes isolated from adult peripheral blood (40, 43, 44). This protocol, when supplemented with TNFα increases Langerin expression and reduces the appearance of DC-SIGN, which is a more characteristic marker of DCs (45–47).

Our goal in the current experiments was to determine what neuropeptide receptors are expressed on moLCs, and which neuropeptides might influence moLC differentiation.

## 2 Results

### 2.1 RNA-Seq and RT-qPCR analyses revealed the expression of NPR1, SORT1, CALCRL and RAMP1 receptors in moLCs

**Figure 1:**
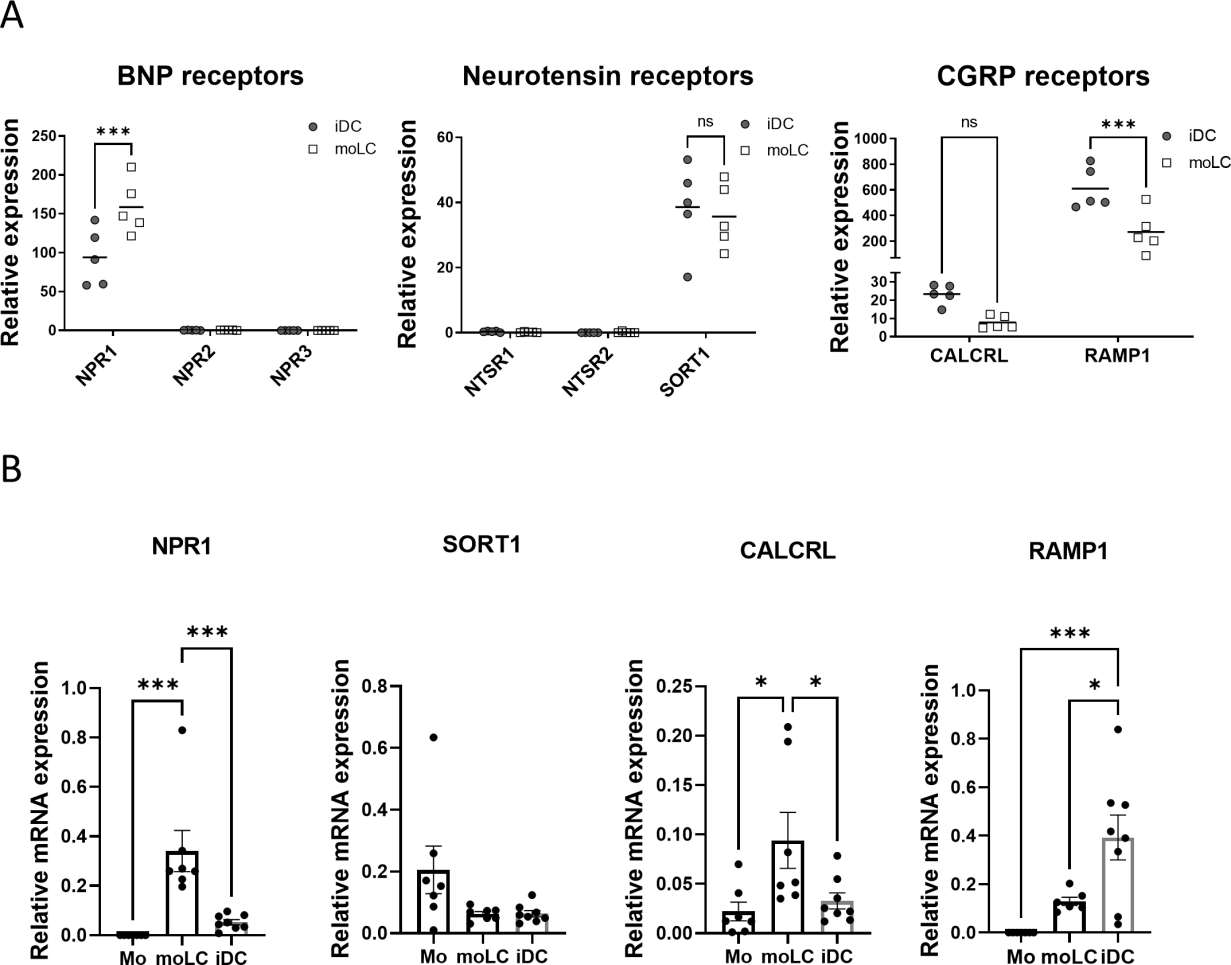
Gene expression of neuropeptide receptors in monocytes, monocyte-derived Langerhans cells (moLCs) and immature dendritic cells (iDCs). NPR1, SORT1, CALCRL, and RAMP1 receptors are expressed in moLCs and iDCs. **(A)** RNA-Seq analysis from moLCs and iDCs based on previously published datasets (46). The symbols represent the number of reads from a single donor, lines mark mean expression levels from all donors. N=5. **(B)** Monocytes were cultured in the presence of GM-CSF, TNF-α, and TGF-β for 5 days supplemented with IL-4 for the first 48 hrs to differentiate moLCs, and cultured in the presence of GM-CSF, IL-4 to differentiate iDCs. Relative mRNA expression of neuropeptide receptors determined with RT-qPCR were compared to PPIA (cyclophilin A). Data is presented as Mean ± SEM. One-way ANOVA with Tukey’s multiple comparison test was used for statistical analysis. Significance was defined as *P<0.05, ***P<0.001, and **** P< 0.0001 N=5–8 CALCRL, Calcitonin receptor-like receptor; NPR1, Natriuretic peptide receptor 1; RAMP1, Receptor activity modifying protein 1; SORT1, Sortilin 1. ns, non-significant.

The first step to examining LCs’ connection with neurons is to explore the neuropeptide receptors in our moLC model (experimental setup, gating strategy and representative dot plots of CD1a and CD207 expression are provided as **Supplementary Figure S1**). We determined the expression of these receptors from RNA-Seq datasets that our workgroup previously published (46), where we used immature DCs (iDCs) as a comparative sample to moLCs (**Figure 1A**). We found four neuropeptide receptors in moLCs and iDCs. NPR1 receptor from the natriuretic peptide family showed significantly higher expression in moLCs compared to the iDCs, while other natriuretic receptors showed no expression. We found that neurotensin receptor genes NTSR1 and NTSR2 are not expressed in any of our cells. SORT1, the third gene of the neurotensin receptor family and CALCRL, the gene of the CGRP receptor showed no difference between iDCs and moLCs and their expression levels were low. The expression of CGRP co-receptor gene RAMP1 was significantly higher in iDCs compared to moLCs.

To validate our RNA-Seq results we next measured mRNA expression with RT-qPCR (**Figure 1B**) on monocytes as well as on moLCs and iDCs differentiated from them. Supporting our RNA-Seq data we found that NPR1 mRNA expression was significantly higher in moLCs than in iDCs or monocytes. SORT1 was more expressed in monocytes, but after differentiation, its expression decreased in each cell type. MoLCs had the highest expression of CALCRL, but RAMP1 was more expressed in iDCs.

### 2.2 MoLCs differentiated in the presence of NPR1 agonists showed increased expression of the surface markers CD207 and CD1a

**Figure 2:**
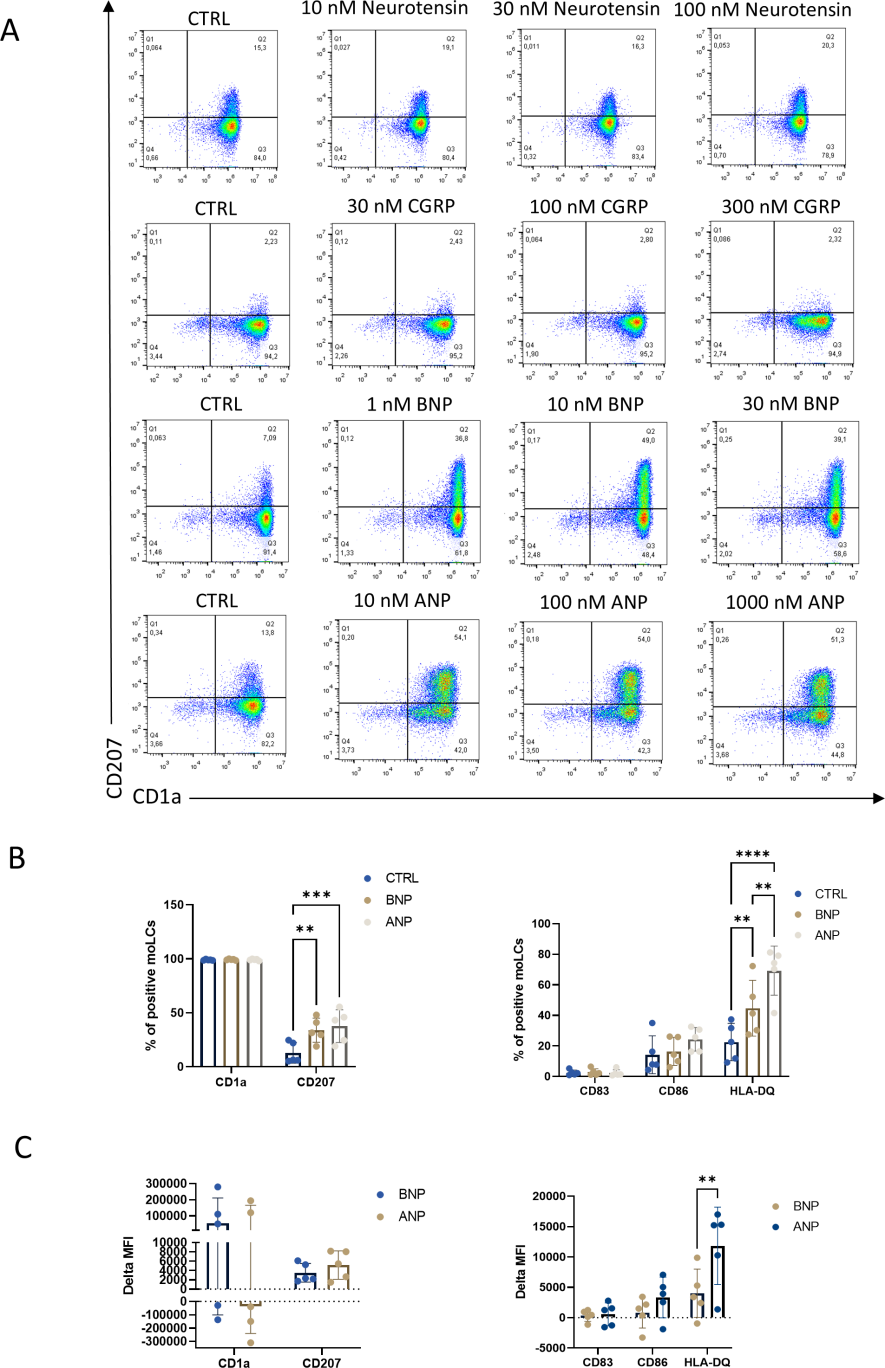
BNP and ANP treatment increased CD1a and CD207 expression after 5 days of differentiation to monocyte-derived Langerhans cells (moLCs). **(A)** Representative density plot of LC markers expression CD1a and CD207 from moLCs as determined by flow cytometry. Monocytes were cultured in the presence of GM-CSF, TNF-α, and TGF-β for 5 days supplemented with IL-4 for the first 48 hrs to differentiate moLCs, and treated with Neurotensin, CGRP, BNP, and ANP in three different concentrations throughout the differentiation process. **(B)** Percentage of CD1a and CD207 positive cells (left) and percentage of CD83, CD86, and HLA-DQ positive cells (right) differentiated in the presence of 100 nM ANP, 10 nM BNP or vehicle (CTRL). Mean ± SD, N=5. **(C)** Delta MFI of moLC markers CD1a and CD207 and activation markers CD83, CD86 and HLA-DQ compared to the control samples by flow cytometry. Mean ± SD, N=5. ANP, Atrial natriuretic peptide; BNP, B-type natriuretic peptide; CGRP, Calcitonin gene-related peptide; CTRL, Control; MFI, mean fluorescence intensity. **P<0.01, *** P<0.001, **** P< 0.0001.

After we explored the neuropeptide receptors in our moLC model we aimed to investigate the effects these neuropeptides have at three distinct concentrations for each neuropeptide on the differentiation of moLCs (**Figure 2**). We analyzed the specific surface markers of LCs CD207 and CD1a with flow cytometry on day 5 of their differentiation (Experimental setup detailed in **Supplementary Figure S2**). Neurotensin and CGRP applied during the differentiation of the cells from day 0 did not affect these markers (**Figure 2A**; **Supplementary Figure S3**), however, natriuretic peptides (ANP and BNP) had a significant, dose-dependent effect on double-positive cells compared to the control samples. Although both ANP and BNP increased the ratio of double positive cells (**Figure 2B**), they did not affect the maturation marker CD83 and the costimulatory molecule CD86. Both peptides in their intermediate concentration significantly increased the expression of HLA-DQ, although the baseline expression was also relatively high, as expected on professional antigen presenting cells (**Figure 2C**). As the other neuropeptides had no significant effect on the investigated markers in the following experiments we decided to focus on natriuretic peptides.

### 2.3 MoLCs started to express NPR1 on the third day of differentiation

**Figure 3:**
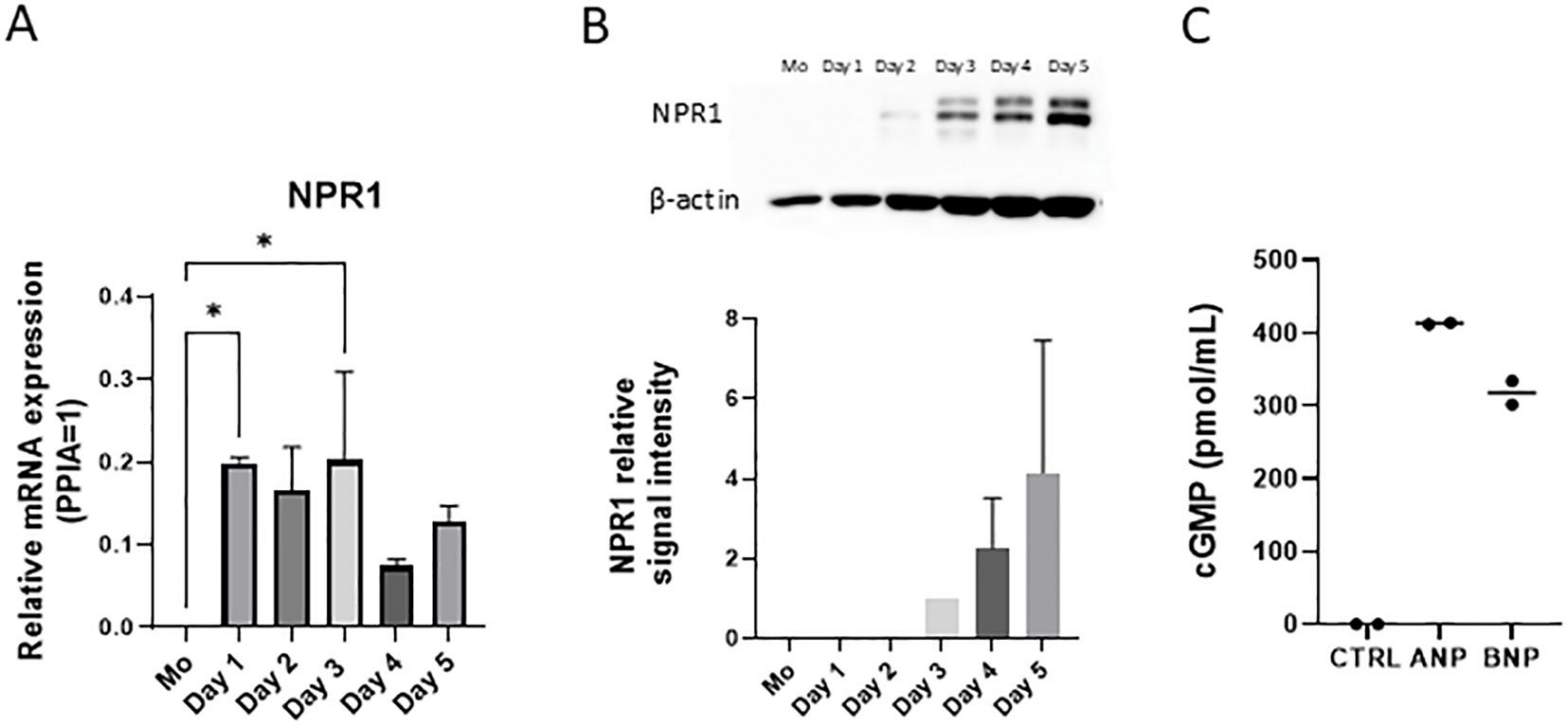
NPR1 is functionally expressed in monocyte-derived Langerhans cells (moLCs) after 3 days of differentiation. **(A)** NPR1 receptor mRNA expression was determined with RT-qPCR. Monocytes were cultured in the presence of GM-CSF, TNF-α, and TGF-β for 5 days supplemented with IL-4 for the first 48 hrs to differentiate moLCs. RNA samples were collected from monocytes on day 0. and every day during the moLCs differentiation. Mean + SD, N=2. **(B)** Western blot analysis of NPR1 expression during moLCs differentiation. Mean + SD, N=3. **(C)** cGMP production was determined by cGMP assay kit after treating moLCs with ANP (100 nM) and BNP (10 nM) for 15 minutes on day 5. N=2 ANP, Atrial natriuretic peptide; BNP, B-type natriuretic peptide; CTRL, Control; NPR1, Natriuretic peptide receptor 1; Mo, monocyte. *P<0.05.

As monocytes did not express NPR1, we used qPCR and western blot to investigate the exact day of differentiation when the cells started to express the natriuretic receptor (**Figures 3A, B**, respectively, experimental setup detailed in **Supplementary Figure S4**). MoLCs started to express NPR1 at the RNA level on the first day of differentiation, while expression on the protein level became detectable only on the third day.

NPR1 has an intracellular guanylate cyclase activity domain and ligand binding results in increased intracellular cGMP levels (48). The effects of BNP and ANP are typically mediated by the cGMP cascade (49). Using an intracellular cGMP assay we could show that both BNP and ANP treatment resulted in increased cGMP levels (**Figure 3C**). Our results show that NPR1 is functionally expressed on moLCs.

### 2.4 NPR1 is widely expressed in the epidermis on keratinocytes and the cell body of LCs

**Figure 4:**
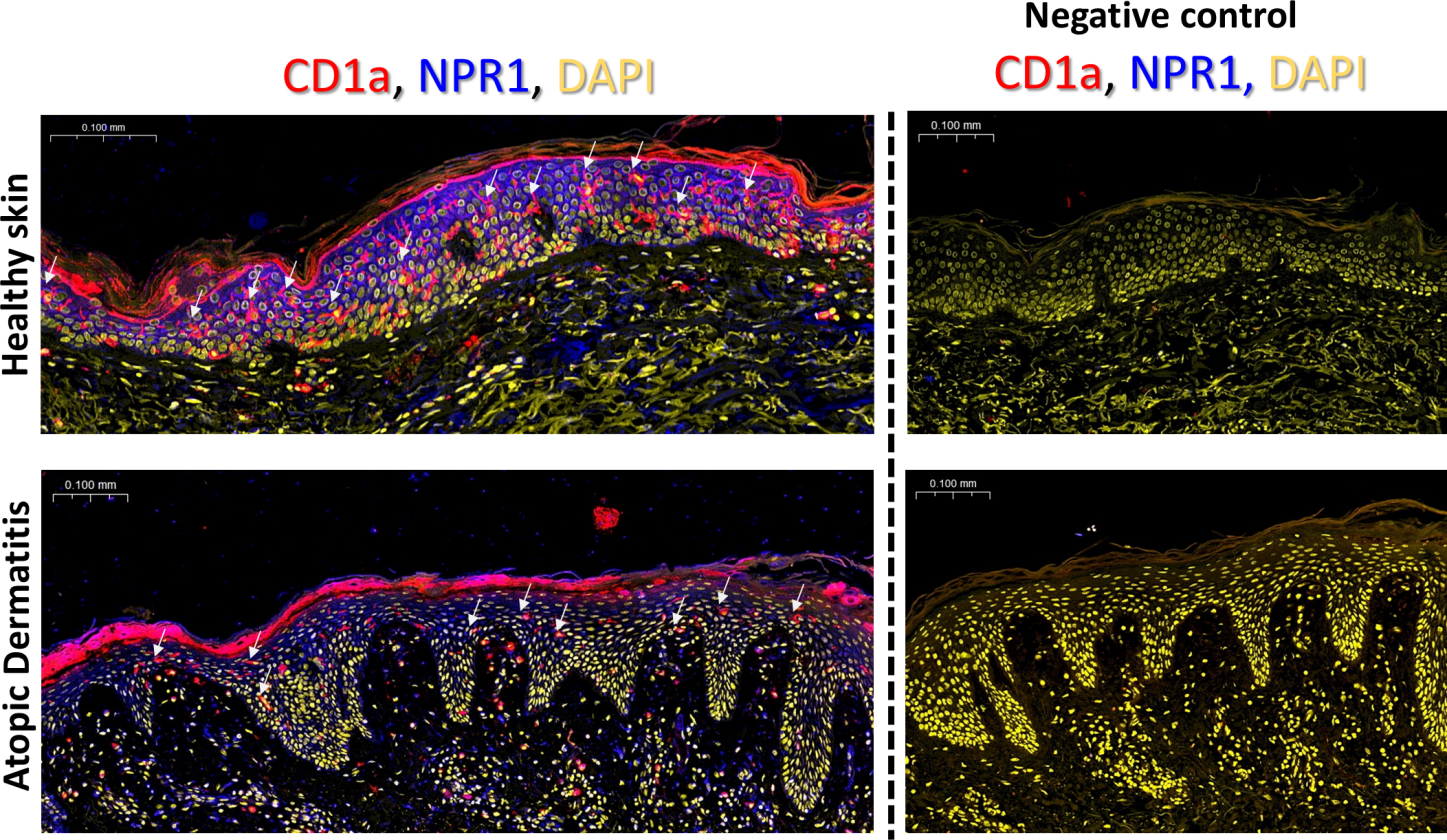
NPR1 is widely expressed *in situ* in the epidermis. Whole slide confocal microscopy images showing the localization of NPR1 and CD1a in the human epidermis of healthy skin and atopic dermatitis lesions. LCs are highlighted with white arrows. Red: CD1a staining, blue: NPR1 staining, yellow: DAPI. The rightmost panel in each row shows the negative control images where primary antibodies were omitted during staining. Two additional donors showed similar results. BNP, B-type natriuretic peptide; CTRL, Control; DAPI, 4′,6-diamidino-2-phenylindole; NPR1, Natriuretic peptide receptor 1.

To determine whether LCs *in situ* in the skin express NPR1 like in our cell model we used whole slide confocal microscopy. We found that skin sections from healthy human skin and lesional AD samples show diffuse epidermal expression of NPR1 (in blue), mostly on keratinocyte plasma membranes. However, in healthy skin, the expression of the natriuretic receptor appears more intense than in AD samples (**Figure 4**). CD1a staining (in red) was used to identify LCs in the epidermis (also highlighted with white arrows).

**Figure 5:**
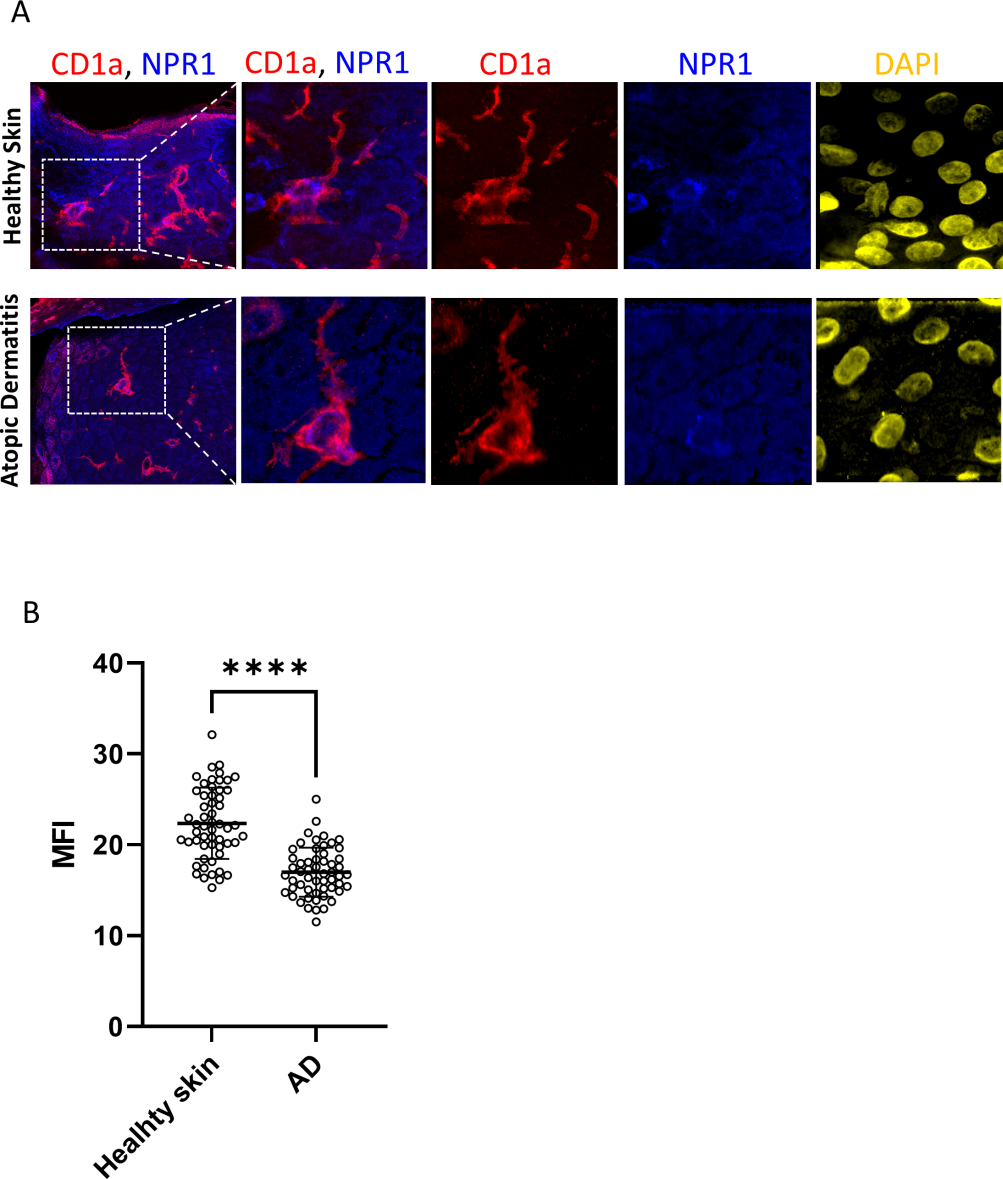
NPR1 receptor is expressed on the Langerhans cells (LCs) cell body but not in the dendrites. **(A)** Confocal and superresolution images showing NPR1 and CD1a coexpression in skin sections from healthy donors and atopic dermatitis lesions focusing on Langerhans cells. NPR1 is localized on the LC cell body but dendrites do not show a positive signal. Red: CD1a staining, blue: NPR1 staining, yellow: DAPI. Field of view is 212.6 x 212.6 µm in the first column, consequent images magnified from the indicated area are 49.4 x 49.4 µm. **(B)** LCs were selected based on CD1a staining and MFI of NPR1 staining on LCs was calculated using ImageJ software. 55 cells were evaluated from three independent donors (N=3) DAPI, 4′,6-diamidino-2-phenylindole; LCs, Langerhans cells; NPR1, Natriuretic peptide receptor 1; ****P< 0.0001.

Due to the widespread expression of NPR1 on keratinocytes we could not determine whether LCs also express this receptor based on lower magnification images. Therefore, we further investigated CD1a^+^ cells at higher magnification with confocal microscopy and saw an intense signal on the cell body of LCs, while the dendrites stayed negative (**Figure 5A**; **Supplementary Videos 1, 2**). According to our findings, some LCs did not give positive signals for NPR1, but we found no difference in the incidence of NPR1 positive and negative LCs between AD and healthy skin. Interestingly, the fluorescence intensity of NPR1 staining on CD1a+ cells shows that LCs of healthy skin expressed more of the receptor compared to those found in AD skin (**Figure 5B**).

### 2.5 MoLCs differentiated in the presence of BNP shift toward a migratory subtype of LCs

**Figure 6:**
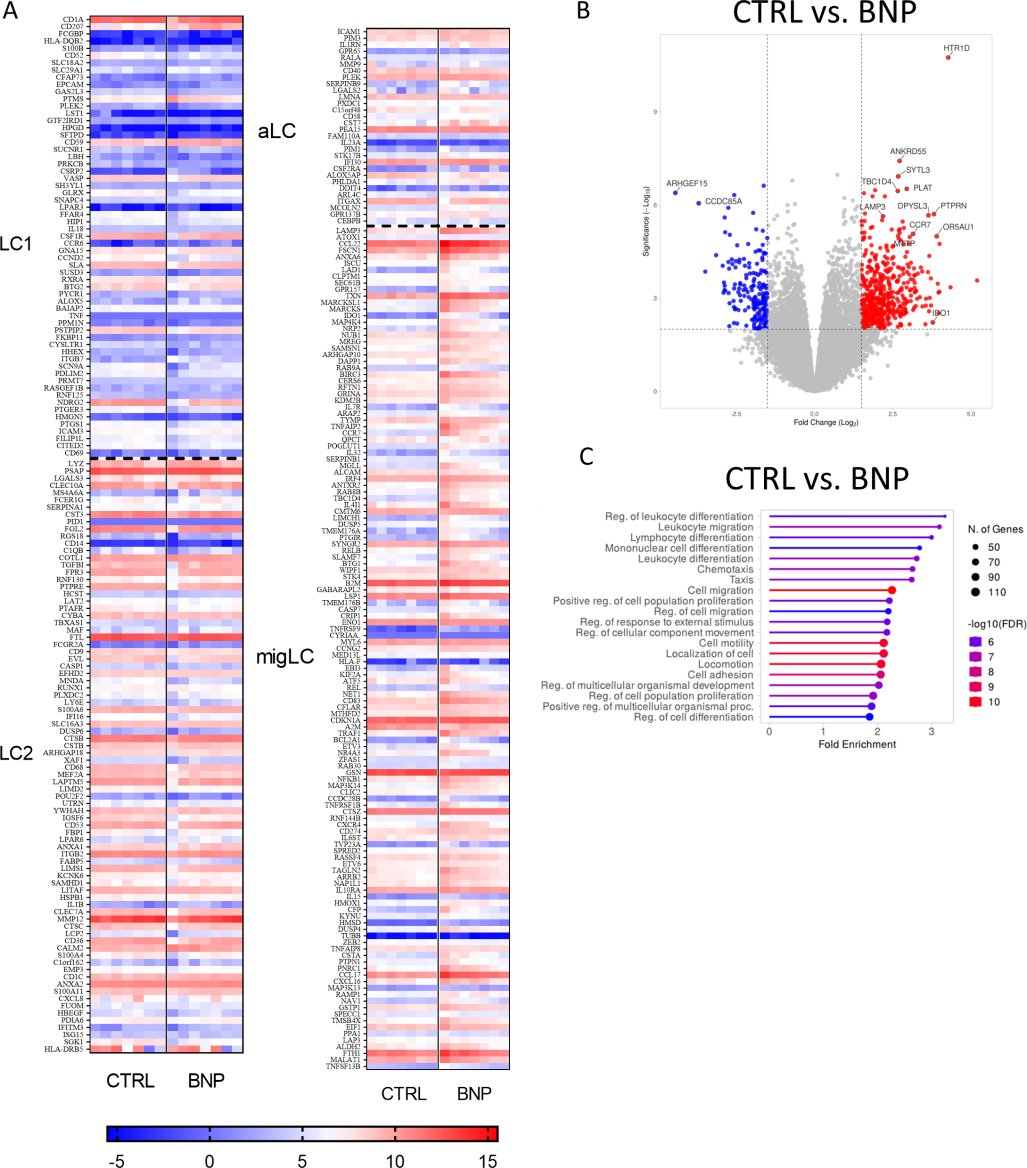
RNA-Seq analysis from BNP treated monocyte-derived Langerhans cells (moLCs) showed a similar gene expression pattern to migratory Langerhans cells (migLCs). Monocytes were cultured in the presence of GM-CSF, TNF-α, and TGF-β for 5 days supplemented with IL-4 for the first 48 hrs to differentiate moLCs and treated with BNP (10 nM) throughout the differentiation process. **(A)** Heatmap analysis of BNP treated moLCs compared to vehicle-treated controls. Cells differentiated in the presence of BNP showed increased expression of genes characteristic of migLC. **(B)** Volcano plot analysis of BNP treated moLCs compared to vehicle-treated controls. Important significantly changed gene symbols are highlighted **(C)** GO annotation of BNP treated moLCs. BNP treatment increases the expression of genes that play a role in T cell interactions. The size of the circle corresponds to the number of induced genes, while the color of the line corresponds to the -log10 of the False Discovery Rate (FDR). N=7 BNP, B-type natriuretic peptide; GO, gene ontology; migLC, migratory Langerhans cell; moLC, monocyte-derived Langerhans cells.

To characterize the effect of NPR1 agonism during the differentiation of moLCs we performed RNA-Seq analysis. As BNP is more widely expressed in the epidermis compared to ANP (15), we applied that ligand during the differentiation of the cells, and characterized our BNP treated moLCs based on the subtype markers established by Liu et al. (50). Our previous investigations showed that moLCs closely resemble the LC2 subtype, which is more likely to be an active state (46). We further investigated the consequences of BNP treatment focusing on the LC subtype markers compared to the control moLCs. Based on the expression of subtype-specific genes BNP applied during the differentiation of the cells did not shift the final phenotype in the direction of either LC1 or activated LC (aLC) subtypes, as shown by the heatmap of marker genes (**Figure 6A**). On the other hand, the expression of migratory markers was higher, which suggests that BNP applied during differentiation can shift the subtype of our moLCs toward a migratory LC (migLC) phenotype. MigLC are prone to express chemokines such as CCL17, CCL22, and chemokine receptors CCR7, and CXCR4 to enhance cutaneous migration towards draining lymph nodes (50–52).

Similar genes were highlighted by volcano plot analysis (**Figure 6B**), which are also related to moLC and T cell interaction. CCR7 and lysosomal-associated membrane protein 3 (LAMP3) impart migratory ability to the cells (53). BNP also upregulates indoleamine 2,3-dioxygenase (IDO1) enzyme expression, which takes part in the tryptophan metabolism associated to T cell regulation, while microsomal triglyceride transfer protein (MTTP) connects to the CD1 molecules’ lipid antigen presentation for T cells (54). We also tested the ability of moLCs differentiated in the presence of BNP to induce T cell proliferation, and found no significant difference was observed compared to control moLCs (**Supplementary Figure S5**).

Gene set enrichment analysis (**Figure 6C**) of differentially expressed genes (DEGs) showed that the most significantly upregulated pathways between cells differentiated in the presence of BNP compared to control cells were related to Cell migration, Cell motility, Localization of cell, Locomotion and Cell adhesion, as well as Lymphocyte, Mononuclear cell and Leukocyte differentiation.

## 3 Discussion

In this study, we investigated the expression of neuropeptide receptors and the effects of their ligands on the differentiation of moLCs. We found that of the investigated neuropeptides ANP and BNP had the most significant effects on the differentiation and phenotype of the cells. Although moLCs were also found to express other neuropeptide receptors (SORT1, CALCRL, RAMP1; **Figure 1**), only ligands of NPR1 increased the expression of CD207 (**Figure 2A**; **Supplementary Figure S1**). SORT1 expression has also been shown on murine fetal-skin DCs, where neurotensin downregulated the expression of proinflammatory cytokines, but had little effect on LPS-induced activation (55); however these cells also express NTR1, and it is unclear how much of this effect is dependent on which receptor. As SORT1 is widely expressed in most tissues (56), and is mostly involved in the transport of proteins between the trans-Golgi network and other compartments (57), it is probable that its main function in these cells is not solely as a receptor for neurotensin. CGRP has previously been reported to have profound impacts on LC function, as it inhibits the increased antigen presentation caused by LPS-induced activation of murine LCs (58). Interestingly, in a human moLC model similar to ours CGRP activates NF-κB and increases CD207 expression, and this resulted in decreased HIV transmission to autologous T cells (59). Notably, in this model the expression of CD207 is lower than in our own, which might explain why we didn’t see a similar effect of CGRP (**Figure 2A**; **Supplementary Figure S1**). NPR1 expression has been proven on other immune cells including DCs, macrophages and thymocytes (60–62), and its transcript can be identified in murine Langerhans cells (63, 64). In macrophages and thymocytes activation of this receptor leads to decreased NO production in the former and decreased proliferation in the latter. DCs produce ANP which acts in an autocrine fashion, and inhibition of NPR1 on the cells primes them to preferentially induce regulatory T cells (62). Contrasting our findings to previous single cell sequencing data from human skin (32), a similar expression pattern to what we described on moLCs can be found on cDC2 langerin positive cells. Epidermal Langerhans cells in their dataset did not express NPR1, but did show some expression of NPR2 and NPR3. Of the other neuropeptide receptors LCs showed expression of SORT1, RAMP1 and CALCRL (although, interestingly at much lower levels than what is expressed in cDC2 cells, and displaying large inter-donor variability). In our previous work (46), we found that moLCs most closely resemble the LC2 subset established by Liu et al. (50), which shows significant overlap with the cDC2 langerin positive cells described previously by Bertram et al. Indeed, there is some debate in the literature between the two groups whether the LC2 subset is in fact the same cDC2 cell population, or whether it should be considered a type of cDC2 cell (33, 65). Nevertheless, our transcriptomic and phenotypic analyses show that moLCs generated using our differentiation protocol differ substantially from conventional monocyte-derived dendritic cells and are more aligned with Langerhans cell-like profiles (46).

Both ANP and BNP increased the expression of CD207 and HLA-DQ on moLCs (**Figure 2B**). Langerin, a specific C-type lectin receptor also known as CD207, is one of the cells’ main markers (37). Langerin is also involved in antiviral mechanisms and has an important role during HIV-1 infection by binding and internalizing the virus into Birbeck granules, thus inhibiting viral transmission to T cells (37, 66, 67). Similarly to NPR1 agonists, TRPV4 activation also induced similar changes in CD207 expression (46). Although it is commonly used as a marker, langerin is not indispensable for LC functions, as LCs from langerin KO mice showed no deficit in antigen capture, presentation, migration or maturation, nor were they more suspectable to skin-tropic microorganisms (68). The increase in HLA-DQ expression is not necessarily a consequence of maturation of these cells, as LCs *in situ* express relatively high levels of HLA-DQ, which is instrumental in promoting tolerance in tissue resident memory T cells (69).

In order to gain a deeper insight into the role of NPR1 on these cells we first wished to identify when the receptor first appears during their differentiation, as previous reports showed that monocytes did not express NPR1 (70, 71). Supporting these findings, we detected NPR1 mRNA in iDCs (**Figure 1A**), but not in monocytes (**Figures 3A, B**). In moLCs the transcript is detectable from the first day of differentiation (**Figure 3A**), while on the protein level it appears only on day 3 (**Figure 3B**). NPR1 activation typically results in an increase of intracellular cGMP (48, 71), which we could detect for both ANP and BNP applied at nanomolar concentrations (**Figure 3C**), showing that the receptor is functional on a molecular level.

We next wished to determine whether NPR1 is also found on LCs *in situ*, and not only in our moLC model. NPR1 has been reported to be widely expressed in the epidermis, as keratinocytes in all layers express the receptor, mainly along the plasma membrane, and this is further increased in atopic skin (15). Similar to the above results we also found abundant NPR1 expression in both healthy and atopic skin, although in our AD skin samples this appeared to be lower compared to the healthy control (**Figure 4**, in blue). To identify LCs in the epidermis we co-stained CD1a on these sections (in red), and found that the number of LCs greatly decreased in lesional atopic skin. Previous findings have reported increased (72–74), unchanged (75) and lower (76–79) LC numbers in lesional AD skin, although all such investigations were limited to a small number of samples (as were ours). An increase in the number of CD1a^+^ cells in the dermal compartment has also been reported (74), which can be explained by the exodus of these cells because of the inflammatory milieu.

Due to the high level of NPR1 expression on keratinocytes confocal images were captured to determine whether LCs also express this receptor, or if their apparent positivity could be attributed to the presence of surrounding cells. We found that in both healthy and AD skin the cell bodies of LCs were positive, while their dendrites did not stain for the receptor (**Figure 5A**; **Supplementary Video 1**). Interestingly, the expression of NPR1 specifically on the cell bodies of LCs was significantly lower in AD skin than in healthy skin. This might be because of higher BNP levels that are characteristic of AD skin (15), which might result in the downregulation of NPR1 in epidermal cells.

To gain a deeper understanding of the effects of NPR1 agonism during the differentiation of moLCs we performed bulk RNA-Seq analysis (**Figure 6**). In these experiments we chose to apply BNP, as it is the more skin-relevant ligand of the receptor (15). BNP is closely associated with itch sensation, as toxin-mediated ablation of responding neurons led to selective loss of behavioral responses to itch-inducing agents (80). In multiple animal models of itch BNP levels have been found to be increased (15, 81, 82). Further, in a murine AD model induced by the vitamin D analogue MC903, BNP-KO mice developed less severe lesions and showed decreased inflammation compared to wild type mice (83). MoLCs differentiated in the presence of BNP showed a pronounced shift towards the migratory LC subtype first characterized by Liu et al. (50) (**Figure 6A**). Supporting this shift volcano plot analysis highlighted the increased expression of LAMP3 and CCR7, two genes which are important in increasing cellular motility and homing towards lymph nodes, respectively, as well as MTTP, which is instrumental in loading neutral lipids onto class one CD1 molecules such as CD1a (**Figure 6B**). Gene set enrichment analysis also resulted in pathways associated with increased cell motility and migration (**Figure 6C**). These changes, combined with the increased expression of IDO1 and the lack of increase in positive costimulatory molecules point to moLCs that are migrating to lymph nodes, where they are likely to initiate tolerogenic responses in naïve T cells.

## 4 Materials and methods

### 4.1 Isolation of monocytes and differentiation of moLCs and moDCs

Buffy coats enhanced with heparinized leukocytes were taken from healthy volunteers. Both the Head of the National Transfusion Service and the Regional and Institutional Ethics Committee of the University of Debrecen’s Faculty of Medicine (Debrecen, Hungary) approved the procedure, as well as the Regional Blood Center of the Hungarian National Blood Transfusion Service (Debrecen, Hungary; approval number: OVSZK 3572-2/2015/5200). PBMCs were collected from human blood samples using gradient centrifugation. To isolate monocytes, we used anti-CD14-conjugated microbeads (Miltenyi Biotech, Bergisch Gladbach, Germany). CD14+ monocytes were cultured in RPMI 1640 medium (Sigma-Aldrich, St. Louis, MI, USA) containing 10% heat-inactivated FBS, 10% HEPES, 50 mM 2-Mercaptoethanol, 1% penicillin (all from Sigma-Aldrich). The cells were plated in 12-well tissue-culture plates (Techno Plastic Products, Trasadingen, Switzerland) at 1x10^6^ cells/ml. To induce the differentiation of moLCs, we supplemented the media with GM-CSF 200 ng/mL (Gentaur Molecular Products, London, UK) TGF β (10 ng/mL), TNFα (20 ng/mL), and IL-4 (20 ng/mL for the first 48 hours) for 5 days at 37°C (the other cytokines from PeproTech, Rocky Hill, NJ, USA). MoLCs were treated with Neurotensin (10 nM, 30 nM, 100 nM), CGRP (30 nM, 100 nM, 300 nM) (Sigma-Aldrich), BNP (1 nM, 10 nM, 30 nM), and ANP (10 nM, 100 nM, 1000 nM ANP) (Bio-techne, Minneapolis, MN, USA) on day 0, day 2 and day 4.

Dendritic cells were differentiated from using well established protocols (84). RPMI 1640 media (Sigma-Aldrich) was supplemented with 10% heat-inactivated FBS, 10% HEPES, 50 mM 2-Mercaptoethanol, 1% penicillin (all from Sigma-Aldrich) as well as GM-CSF (80 ng/ml) (Gentaur Molecular Products) and IL-4 (20 ng/ml) (PeproTech) at a 1x10^6^ cells/ml density in a 24-well plate, and cells were cultured for 5 days at 37°C.

### 4.2 RNA-Seq method

MoLC samples were collected on day 5 for high throughput mRNA Illumina sequencing analysis. RNA integrity was measured with Agilent BioAnalyzer using an Eukaryotic Total RNA Nano Kit (Agilent Technologies, Waldbronn, Germany). The applicable RNA integrity number was 7 for the library preparation. RNA using Ultra II RNA Sample Prep kit (New England BioLabs Inc., Ipswich, MA, USA) was used for the libraries. An Illumina NextSeq 500 instrument (Illumina, Inc., San Diego, CA, USA) was used for sequencing. Statistical analysis was performed with StrandNGS software (www.strand-ngs.com). Library preparation, sequencing and primary data analysis were carried out at the Genomic Medicine and Bioinformatics Services Laboratory of the University of Debrecen. Further evaluation was made using the Galaxy web platform (usegalaxy.org). Volcano plots were generated with the help of the VolcaNoseR web app (85). Gene set enrichment analysis was performed using the ShinyGO web app (85).

### 4.3 Measurement of intracellular cGMP levels

MoLCs were collected on day 5 of differentiation and BNP was added to the cells for 15 minutes. After that cells were washed three times with phosphate-buffered saline (PBS) and resuspended in Cell Lysis Buffer from the kit. Cells were frozen at -20 °C and after repeating the thaw cycle we centrifuged at 600 x g for 10 minutes at 2-8 °C to remove the cellular debris. We used 200 µl cell lysate to perform the assay per the manufacturer’s instructions (cGMP Assay, R&D Systems, Wiesbaden, Germany). An EnVision 2105 Multimode Plate Reader (Perkin Elmer, Waltham, MA, USA) was used to quantify cGMP concentration.

### 4.4 Immunofluorescence

Paraffin-embedded skin samples were sectioned using a microtome (Leica Biosystems, Nussloch, Germany) into 6 µm sections. After deparaffinization and rehydration, citrate buffer pH 6.1 at 90°C for 20 minutes was used for antigen retrieval. Sections were washed with PBS + 0.05% Tween 20, then blocked with PBS+1% BSA+0.5% Triton X-100 for 20 minutes. Samples were stained with primary antibodies against NPR1 (R&D systems), and CD1a (Thermo Fisher Scientific, Waltham, MA, USA) at 4°C overnight. After thorough washing sections were incubated with fluorophore-conjugated secondary antibodies for 1 hour at room temperature, nuclei were counterstained using 4’-6-diamidino-2-phenylindole (DAPI, Thermo Fisher Scientific) and finally samples were mounted with Fluoromount Aqueous Mounting Medium (Sigma-Aldrich) The complete sections were scanned with a Pannoramic Confocal digital fluorescence slide scanner (3DHISTECH Kft., Budapest, Hungary) and cells were annotated in Slide Viewer software (3DHISTECH Kft). Specific regions of interest were scanned at superresolution with a Zeiss LSM 880 AiryScan (Carl Zeiss-AG, Jena, Germany). The MFI of NPR1 on Langerhans cells was calculated using Fiji software (86).

### 4.5 RNA isolation, reverse transcription, and quantitative “Real-Time” polymerase chain reaction PCR

Total RNA from moLCs was isolated using guanidine isothiocyanate reagent (TRIzolate Reagent, Thermo Fisher Scientific) according to manufacturer’s protocol, and the isolated RNA was quality-checked with a Nanodrop-1000 Spectrophotometer (Thermo Fisher Scientific). Genomic DNA was removed from samples using DNase treatment (DNase I enzyme), after which RNA was reverse transcribed to cDNA using a High Capacity cDNA Reverse Transcription Kit (Thermo Fisher Scientific).

PCR amplification was performed using TaqMan primers and probes (Thermo Fisher Scientific, NPR1: Hs01099745_m1, SORT1: Hs00361760_m1, CALCRL: Hs00907738_m1, RAMP1: Hs00195288_m1) and TaqMan Gene Expression Master Mix (Thermo Fisher Scientific) on a LightCycler 480 (Roche Life Science, Penzburg, Germany) instrument. The amount of the transcripts was normalized to those of the relevant housekeeping gene using the ΔCT method.

### 4.6 Flow cytometry analysis

MoLCs were collected on day 5, washed with FACS buffer containing PBS 2 (v/v) % heat-inactivated FBS and 2 mM EDTA (pH 7.4) and stained with the following antibodies CD1a-Phycoerythrin (PE), CD207-Allophycocyanin (APC), CD83-Fluorescein Isothiocyanate (FITC), (all from BioLegend, San Diego, CA, USA), CD86-PE (R&D Systems), and HLA-DQ-APC (Thermo Fisher Scientific) for 20 minutes on ice.

Stained cells were measured with an ACEA NovoCyte 2000R cytometer (ACEA Biosciences, San Diego, CA USA) and results were analyzed with FlowJo 10.8.1 software (BD Biosciences, Franklin Lakes, NJ, USA).

### 4.7 Western blotting

Cells were collected in a detergent mix to lyse cells, and proteins (30 µg) were loaded to SDS-PAGE (10% gels) and transferred to nitrocellulose membranes (Bio-Rad, Hercules, CA, USA). Membranes were blocked with 5% non-fat milk powder solution and incubated with primary antibody against NPR1 overnight at 4°C. A horseradish peroxidase-conjugated goat anti-rabbit IgG was used as a secondary antibody to detect the primary antibody (Thermo Fisher Scientific), and protein bands were visualized with SuperSignal West Pico Chemiluminescent Substrate-enhanced chemiluminescence kit (Thermo Fisher Scientific) using an Azure c300 imaging system (Azure Biosystems, Dublin, CA, USA).

### 4.8 Statistical analysis

Individual statistical analyses were performed using GraphPad Prism 9.1.2. for Windows (GraphPad Software, San Diego, CA, USA). Two-sample comparison of 2 groups, two-sided, unpaired Student’s t-test, comparing three or more groups analyzed by one-way ANOVA followed by Tukey or Dunnet post-analysis. The differences were considered statistically significant for P < 0.05.

## Data Availability

The datasets generated for this study can be found in the Gene Expression Omnibus repository hosted at the National Library of Medicine, under accension number GSE291916.
