# Supplementary material for: Natriuretic peptides modulate monocyte-derived Langerhans cell differentiation and promote a migratory phenotype
Source: Front Immunol. 2025 Jun 9;16:1593141. doi: 10.3389/fimmu.2025.1593141 (PMC12183070; doi:10.3389/fimmu.2025.1593141)
Supplement: Supplementary file 1 [file DataSheet1.pdf]

Supplementary Material

1.1 Supplementary Figures

A

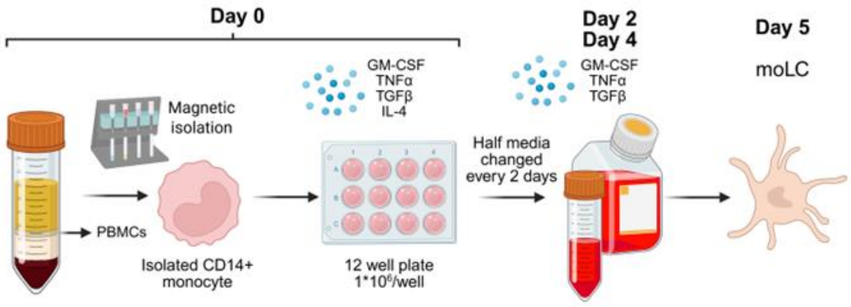

B

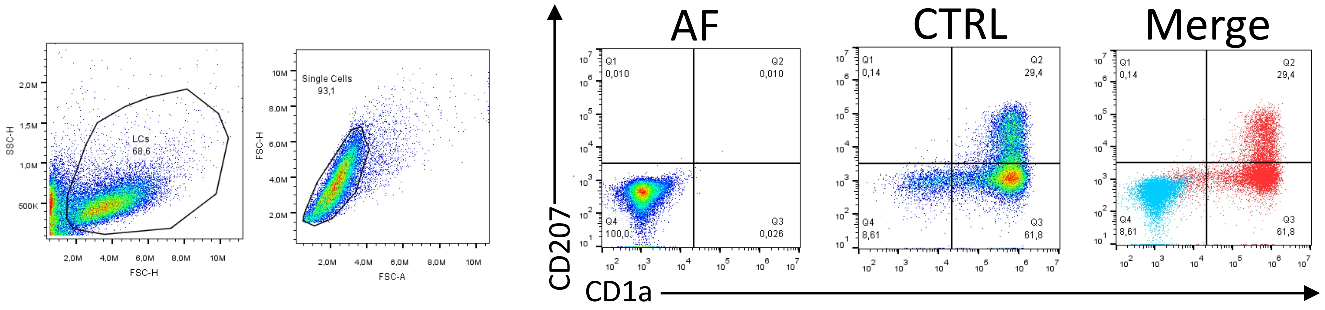

Supplementary Figure 1: Experimental setup of moLCs differentiation, gating strategy, and representative dot plots

(A) Schematic representation of the experimental setup of moLCs differentiation. **Created in BioRender. Horváth, D. (2025)** https://BioRender.com/0j1q3k8 **(B)** Flow cytometry gating strategy was used to define the moLCs population. MoLCs were gated based on FSC-H and SSC-H, and single cells were selected from the FSC-A versus FSC-H dot plot. The quadrant in the density plot of CD1a and CD207 markers was adjusted to the autofluorescence of unlabeled cells.

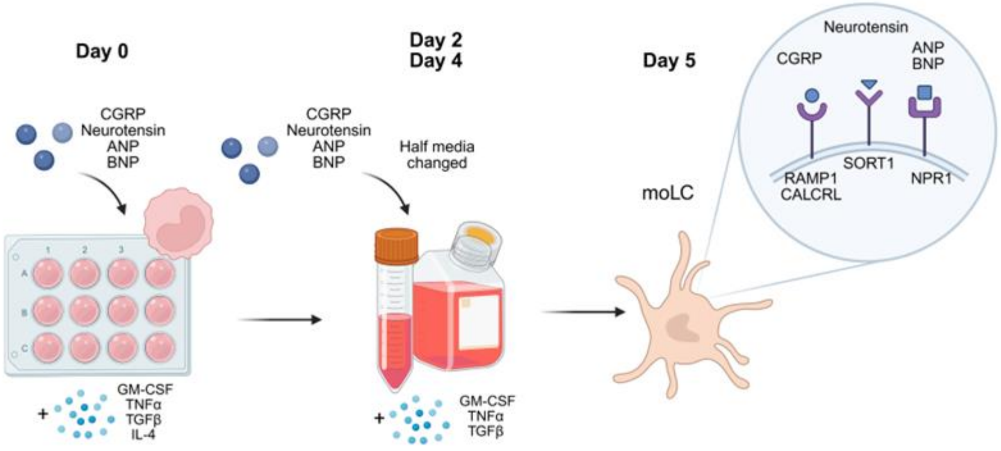

Supplementary Figure 2: Experimental setup of moLCs differentiation and neuropeptides treatment

Schematic representation of the experimental setup of moLCs differentiation, neuropeptides treatment and ligand-receptor pairs of neuropeptide receptors (in insert). **Created in BioRender. Horváth, D. (2025)** https://BioRender.com/kg85mcj

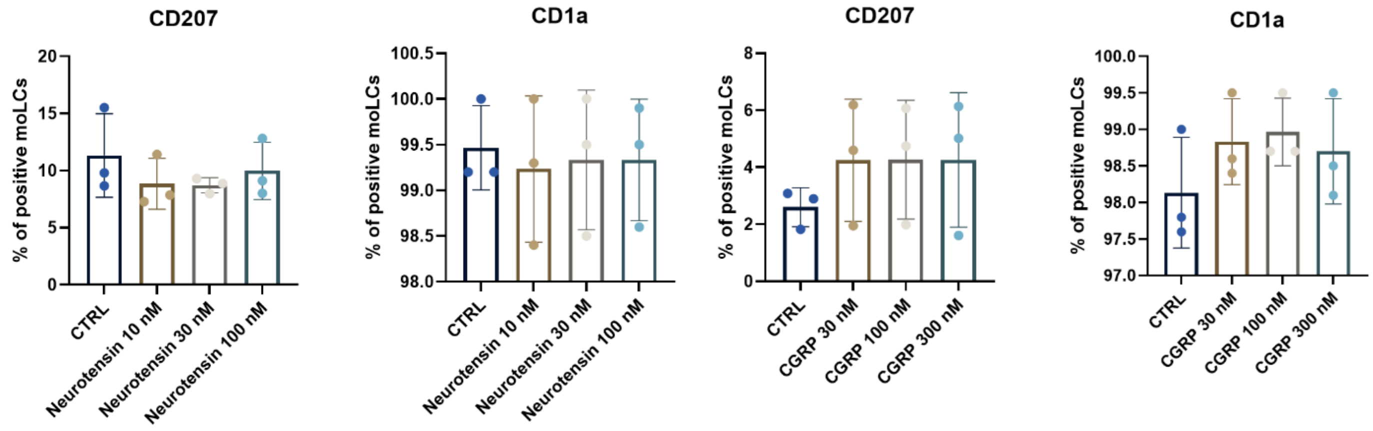

Supplementary Figure 3: Neurotensin and CGRP treatment did not change the differentiation of monocyte-derived Langerhans cells (moLCs).

Percentage of CD1a and CD207 positive cells compared to the control samples by flow cytometry. Monocytes were cultured in the presence of GM-CSF, TNF-α, and TGF-β for 5 days supplemented with IL-4 for the first 48 hrs to differentiate moLCs, and treated with Neurotensin, CGRP applied at three different concentrations throughout the differentiation process. N=3 CGRP, Calcitonin gene-related peptide; CTRL, Control.

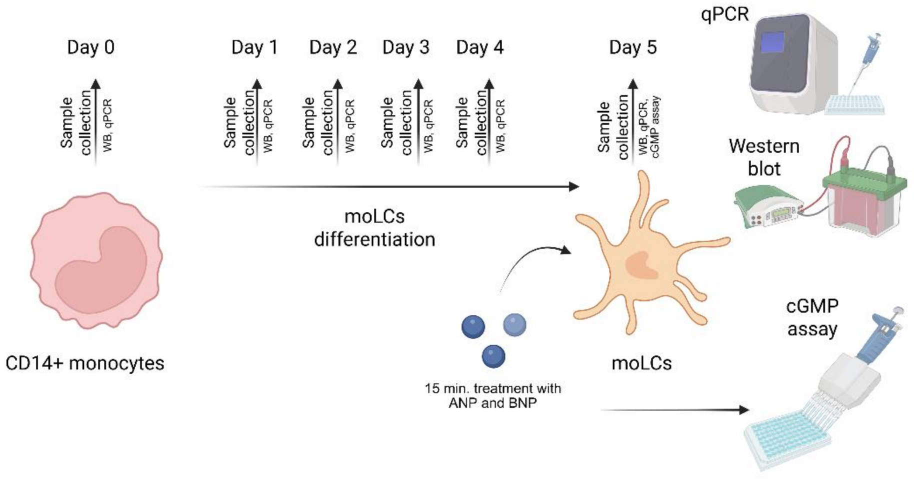

Supplementary Figure 4: Experimental setup of moLCs differentiation and sample collection for qPCR, Western blot, and cGMP assay.

Created in BioRender. Horváth, D. (2025) https://BioRender.com/oexgpio

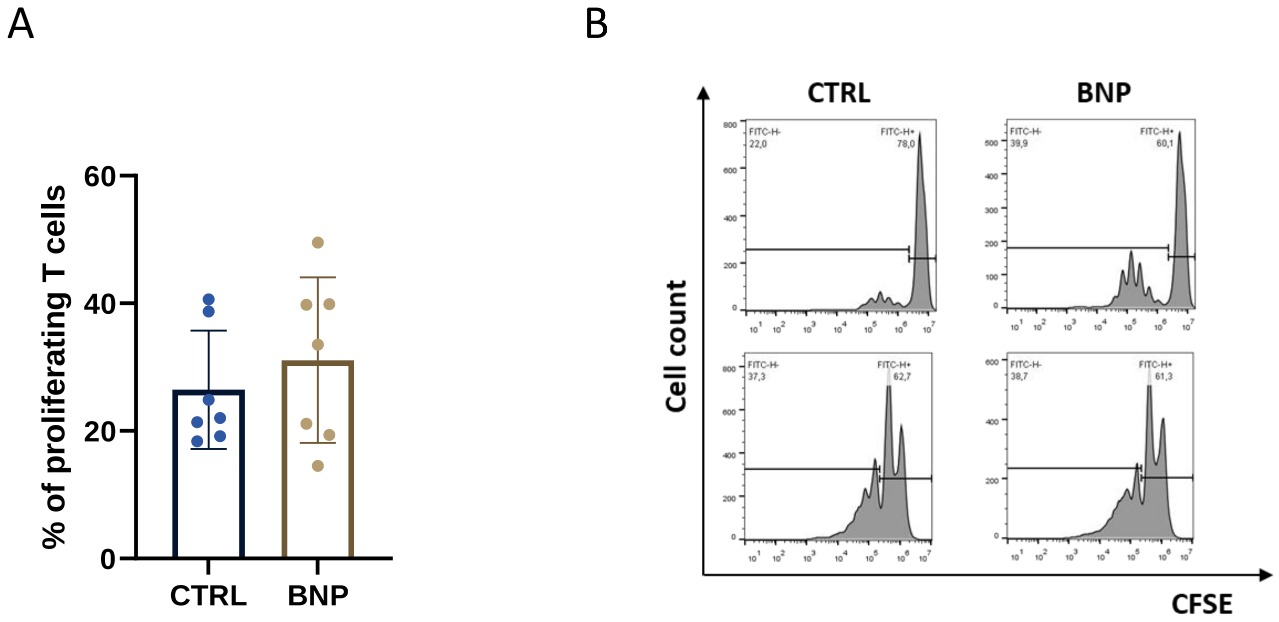

Supplementary Figure 5: BNP does not significantly influence T cell proliferation induction ability of moLCs.

(A) Percentage of proliferating T cells after made a coculture with moLCs and measured the CFSE intensity by flow cytometry on day 5. Monocytes were cultured in the presence of GM-CSF, TNF-α, and TGF-β for 5 days supplemented with IL-4 for the first 48 hrs to differentiate moLCs, and treated with 10 nM BNP or vehicle (CTRL) throughout the differentiation process. Naïve CD4+ T cells were isolated with CD4+ magnetic beads and were cocultured with fully differentiated moLCs for 5 days. **(B)** Representative histograms of CFSE intensity from T cell and moLC coculture. N=7 BNP, B-type natriuretic peptide; CFSE, Carboxyfluorescein succinimidyl ester; CTRL, Control;

Supplementary Videos: Langerhans cells (LCs) expressed the NPR1 receptor on their cell body but not on the dendrites in healthy and atopic skin samples.

Representative 3D videos by confocal microscopy showing NPR1 and CD1a coexpression focusing on Langerhans cells in healthy and atopic skin samples generated by Fiji software. Red: CD1a staining, blue: NPR1 staining, yellow: DAPI. DAPI, 4′,6-diamidino-2-phenylindole; NPR1, Natriuretic peptide receptor 1.

1.2 Supplementary materials and methods

T cell proliferation

Naïve CD4+ T cells were isolated from human buffy coat using the Naïve CD4+ Isolation Kit (Miltenyi Biotec), following the manufacturer's protocol. CD4+ T cells were stained with 0.5 µM carboxyfluorescein succinimidyl ester dye (CFSE) (Thermo Fisher Scientific) and made a coculture with moLCs in 1:10 ratio using round bottom 96 well plate. The coculture RPMI 1640 medium is supplemented with 1 µg/ml anti-human CD3 mAb (BD-Biosciences, San Jose, CA, USA). Cells in the coculture were collected at day 5 and CFSE intensity was measured by flow cytometry using an ACEA NovoCyte 2000R cytometer (ACEA Biosciences).
